# Expression and prognosis analysis of *TET* family in acute myeloid leukemia

**DOI:** 10.18632/aging.102928

**Published:** 2020-03-25

**Authors:** Tingjuan Zhang, Yangli Zhao, Yangjing Zhao, Jingdong Zhou

**Affiliations:** 1 Department of Hematology, Affiliated People’s Hospital of Jiangsu University, Zhenjiang, Jiangsu, People’s Republic of China; 2 Zhenjiang Medical School, Nanjing Medical University, Zhenjiang, Jiangsu, People’s Republic of China; 3 Zhenjiang Clinical Research Center of Hematology, Zhenjiang, Jiangsu, People’s Republic of China; 4 The Key Lab of Precision Diagnosis and Treatment in Hematologic Malignancies of Zhenjiang, Zhenjiang, Jiangsu, People’s Republic of China; 5 Department of Bioinformatics, School of Biomedical Engineering and Informatics, Nanjing Medical University, Nanjing, Jiangsu, People’s Republic of China; 6 Department of Immunology, Key Laboratory of Medical Science and Laboratory Medicine of Jiangsu Province, School of Medicine, Jiangsu University, Zhenjiang, Jiangsu, People’s Republic of China

**Keywords:** TET, expression, prognosis, HSCT, AML

## Abstract

*TET* family members (*TETs*) encode proteins that represent crucial factors in the active DNA demethylation pathway. Evidence has proved that *TET2* mutation is associated with leukemogenesis, drug response, and prognosis in acute myeloid leukemia (AML). However, few studies revealed the *TETs* expression and its clinical significance in AML. We conducted a detailed expression and prognosis analysis of TETs expression in human AML cell lines and patients by using public databases. We observed that *TETs* expression especially *TET2* and *TET3* was closely associated with AML among various human cancers. *TET1* expression was significantly reduced in AML patients, whereas *TET2* and *TET3* expression was significantly increased. Kaplan-Meier analysis showed that only *TET3* expression was associated with overall survival (OS) and disease-free survival (DFS) among both total AML as well as non-M3 AML, and was confirmed by another independent cohort. Moreover, Cox regression analysis revealed that *TET3* expression may act as an independent prognostic factor for OS and DFS in total AML. Interestingly, patients that received hematopoietic stem cell transplantation (HSCT) did not show significantly longer OS and DFS than those who did not receive HSCT in *TET3* high-expressed groups; whereas, in *TET3* low-expressed groups, patients that accepted HSCT showed significantly longer OS and DFS than those who did not accept HSCT. By bioinformatics analysis, *TET3* expression was found positively correlated with tumor suppressor gene including *CDKN2B*, *ZIC2*, *miR-196a*, and negatively correlated with oncogenes such as *PAX2* and *IL2RA*. Our study demonstrated that *TETs* showed significant expression differences in AML, and *TET3* expression acted as a potential prognostic biomarker in AML, which may guide treatment choice between chemotherapy and HSCT.

## INTRODUCTION

DNA methylation has contributed to the understanding of the complexities of genomic instability and gene regulation without altering the DNA sequence [1]. Aberrations in DNA methylation status are closely associated with tumor progression and prognosis of patients especially in blood cancers including acute myeloid leukemia (AML) [1, 2]. During malignant transformation, CpG islands in the promoter region of numerous genes become hypermethylated, silencing the expression of suppressor genes, and leading to a loss in the control of cell apoptosis, proliferation, and differentiation [1]. Conversely, hypomethylation of oncogenes enhances the tumorigenic potential of normal cells [1]. The process of DNA methylation controlled by several molecules such as DNA methyltransferases (DNMTs) has been well characterized [3, 4], but the underlying mechanism of demethylation remains to be elucidated. In recent years, Ten-eleven translocation (TET) proteins have been identified and expand the understanding about mechanisms of DNA demethylation [5].

The TET protein family includes TET1, TET2 and TET3, which can modify 5-methylcytosine (5-mC) by oxidation to 5-hydroxymethylcytosine (5-hmC) and further 5-formylcytosine (5-fC) and 5-carboxycytosine (5-caC) [6–8]. *TET* family members (*TETs*) were dysregulated in multiple malignances, and loss-of-function mutations or decreased expression of *TETs* inhibited the DNA demethylation pathway, which prevents the removal of 5mC from genomic DNA [5]. Functional studies have revealed the direct role of *TET2* in blood cancers especially in AML. Cimmino et al reported that restoration of *TET2* reversed aberrant hematopoietic stem and progenitor cell self-renewal in vitro and in vivo, and suppressed human leukemic colony formation and leukemia progression of primary human leukemia patient-derived xenografts [9]. Rasmussen et al indicated that loss of *TET2* in hematopoietic cells lead to DNA hypermethylation of active enhancers and induction of leukemogenesis [10]. *TET2* mutations frequently occur in AML, myelodysplastic syndromes (MDS) and chronic myelomonocytic leukemia (CMML), whereas *TET1* and *TET3* mutations rarely happen [11, 12]. Moreover, *TET2* mutations were important prognostic factors in AML and also predicted response to hypomethylating agents in MDS patients [13]. However, few studies investigated *TETs* expression and its clinical significance in AML [14, 15]. Herein, we determined the clinical significance of *TETs* expression in AML among The Cancer Genome Atlas (TCGA) databases.

## RESULTS

### TETs expression associated with AML among human cancer cell lines

**Figure 1:**
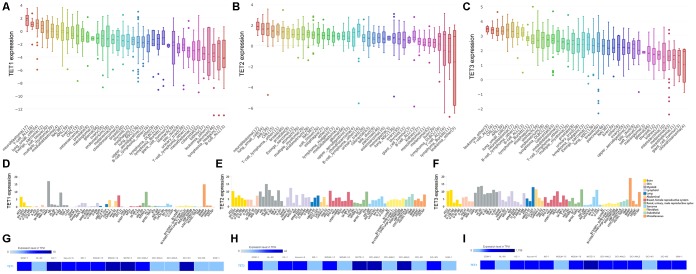
**The expression of *TETs* in human cancer cell lines including AML cell lines**. (**A**–**C**) The expression of *TETs* in human cancer cell lines, analyzing by the Cancer Cell Line Encyclopedia (CCLE) dataset (https://www.broadinstitute.org/ccle). (**D**–**F**) The expression of *TETs* in human cancer cell lines, analyzing by The Human Protein Atlas (HPA) dataset (https://www.proteinatlas.org/). (**G**–**I**) The expression of *TETs* in leukemia cell lines, analyzed by the European Bioinformatics Institute (EMBL-EBI) dataset (https://www.ebi.ac.uk).

By assembling the Cancer Cell Line Encyclopedia (CCLE), we found that *TETs* expression especially *TET2* and *TET3* was highly expressed in AML cell lines among 40 types of human cancer cell lines (Figure 1A–1C). Moreover, The Human Protein Atlas (HPA) also presented that *TET2* and *TET3* expression was also highly associated with myeloid cell lines (Figure 1D–1F). The detailed comparison of *TETs* expression in AML cell lines was assessed by using the European Bioinformatics Institute (EMBL-EBI) website (Figure 1G–1I). In addition, *TET1*/*2*/*3* mutations in human cancer cell lines were given in Supplementary Table 1.

### TETs expression associated with AML patients among human cancers

**Figure 2:**
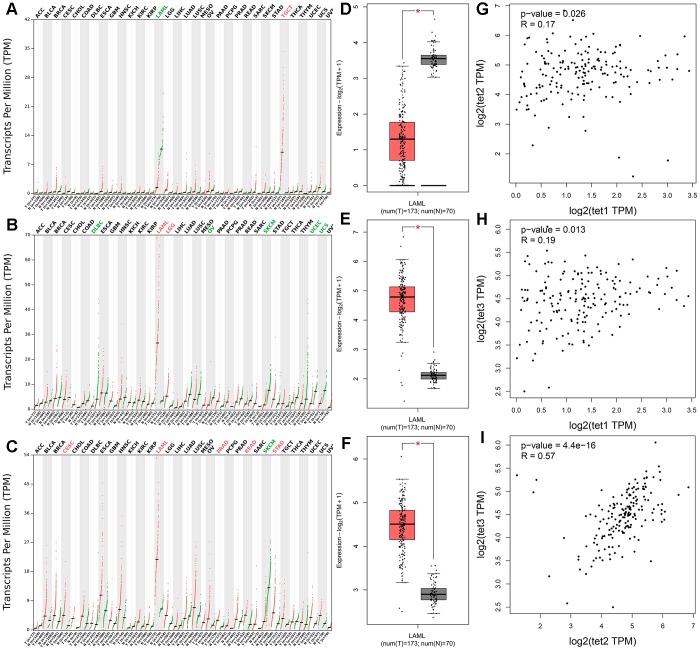
**The expression of *TETs* in human cancers including AML patients.** (**A**–**C**) The expression of *TETs* in pan-cancer analyzed by the Gene Expression Profiling Interactive Analysis (GEPIA) dataset (http://gepia.cancer-pku.cn/). Tumor abbreviations: ACC: Adrenocortical carcinoma; BLCA: Bladder Urothelial Carcinoma; BRCA: Breast invasive carcinoma; CESC: Cervical squamous cell carcinoma and endocervical adenocarcinoma; CHOL: Cholangiocarcinoma; COAD: Colon adenocarcinoma; DLBC: Lymphoid Neoplasm Diffuse Large B-cell Lymphoma; ESCA: Esophageal carcinoma; GBM: Glioblastoma multiforme; HNSC: Head and Neck squamous cell carcinoma; KICH: Kidney Chromophobe; KIRC: Kidney renal clear cell carcinoma; KIRP: Kidney renal papillary cell carcinoma; LAML: Acute Myeloid Leukemia; LGG: Brain Lower Grade Glioma; LIHC: Liver hepatocellular carcinoma; LUAD: Lung adenocarcinoma; LUSC: Lung squamous cell carcinoma; MESO: Mesothelioma; OV: Ovarian serous cystadenocarcinoma; PAAD: Pancreatic adenocarcinoma; PCPG: Pheochromocytoma and Paraganglioma; PRAD: Prostate adenocarcinoma; READ: Rectum adenocarcinoma; SARC: Sarcoma; SKCM: Skin Cutaneous Melanoma; STAD: Stomach adenocarcinoma; TGCT: Testicular Germ Cell Tumors; THCA: Thyroid carcinoma; THYM: Thymoma; UCEC: Uterine Corpus Endometrial Carcinoma; UCS: Uterine Carcinosarcoma; UVM: Uveal Melanoma. Tumor abbreviations showed in black indicated no TETs over- or under-expression, in red color indicated *TETs* overexpression, whereas in green color indicated *TETs* underexpression. (**D**–**F**) The expression of *TETs* in AML analyzed by the GEPIA dataset (http://gepia.cancer-pku.cn/). (**G**–**I**) The correction between *TETs* in AML analyzed by the GEPIA dataset (http://gepia.cancer-pku.cn/).

We further evaluated *TETs* expression in AML patients by using the Gene Expression Profiling Interactive Analysis (GEPIA) dataset including TCGA and the Genotype-Tissue Expression (GTEx) projects. Aberrant expression of all *TETs* members was only observed in AML patients among 33 types of human cancers (Figure 2A–2C). *TET1* expression was significantly reduced in AML patients, whereas *TET2* and *TET3* expression was significantly increased in AML patients (Figure 2D–2F). Moreover, *TET1* expression did not show a significant correlation with *TET2*/*TET3* expression in AML patients, whereas *TET2* expression was positively correlated with *TET3* expression in AML patients (Figure 2G–2I). In addition, *TET1* and *TET3* mutations were identified in none of these AML patients, whereas *TET2* mutation was identified in 8.5% (17/200) of these AML patients.

### Prognostic value of TETs expression in AML

**Figure 3:**
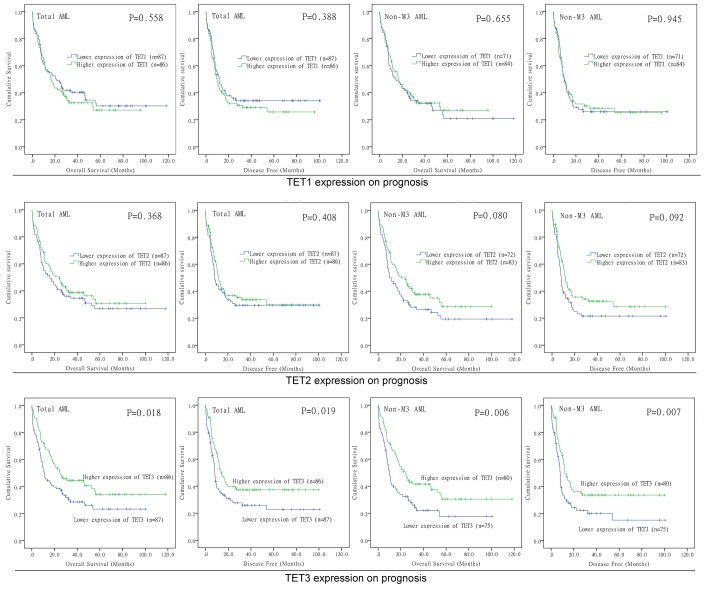
**The impact of *TETs* expression on survival of AML patients.** Kaplan–Meier survival curves of *TETs* expression on overall survival and disease free survival in both chemotherapy and hematopoietic stem cell transplantation groups.

In order to evaluate the prognostic value of *TETs* expression in AML, we further divided these patients into two groups based on median level of *TET1*/*2*/*3* transcript respectively (*TET1*^low^ vs. *TET1*^high^; *TET2*^low^ vs. *TET2*^high^; *TET3*^low^ vs. *TET3*^high^). Based on Kaplan-Meier analysis, we did not observe the significant associations of *TET1* and *TET2* expression with overall survival (OS) and disease-free survival (DFS) among both total AML and non-M3 AML (Figure 3). However, *TET3*^high^ patients showed markedly longer OS and DFS than *TET3*^low^ patients among total AML (Figure 3, *P*=0.018 and 0.019, respectively). Moreover, if French-American-British (FAB)-M3 patients were excluded, patients with high expression of *TET3* also had significantly longer OS and DFS than those with low expression of *TET3* (Figure 3, *P*=0.006 and 0.007, respectively). We next determined the prognostic effect of *TET3* expression in AML by using Cox regression analysis. Both univariate and multivariate analysis showed that *TET3* expression may act as an independent prognostic factor for OS and DFS in total AML (Table 1, *P*=0.011 and 0.026, respectively) and non-M3 AML (Table 2, *P*=0.038 and 0.026, respectively).

**Table 1:** Cox regression analyses of variables for OS and DFS in total AML patients.

**Variables**	**OS**				**DFS**			
	**Univariate analysis**		**Multivariate analysis**		**Univariate analysis**		**Multivariate analysis**	
	**HR (95% CI)**	***P***	**HR (95% CI)**	***P***	**HR (95% CI)**	***P***	**HR (95% CI)**	***P***
*TET3* expression	0.644 (0.445-0.932)	0.020	0.610 (0.416-0.895)	0.011	0.647 (0.447-0.936)	0.021	0.647 (0.441-0.950)	0.026
Age	1.040 (1.027-1.054)	0.000	1.023 (1.007-1.039)	0.005	1.035 (1.022-1.048)	0.000	1.022 (1.007-1.038)	0.005
WBC	1.003 (0.999-1.006)	0.119	1.008 (1.004-1.012)	0.000	1.003 (1.000-1.006)	0.091	1.008 (1.004-1.012)	0.000
Karyotype risk	1.854 (1.465-2.346)	0.000	1.687 (1.236-2.303)	0.001	1.829 (1.448-2.311)	0.000	1.853 (1.398-2.455)	0.000
Treatment regimen	0.551 (0.389-0.780)	0.001	0.398 (0.254-0.623)	0.000	0.615 (0.434-0.871)	0.006	0.476 (0.308-0.734)	0.001
*FLT3* mutations	1.269 (0.869-1.852)	0.217			1.254 (0.859-1.829)	0.241		
*NPM1* mutations	1.220 (0.837-1.778)	0.301			1.268 (0.869-1.848)	0.218		
*CEBPA* mutations	0.913 (0.464-1.796)	0.792			1.053 (0.535-2.073)	0.881		
*DNMT3A* mutations	1.615 (1.104-2.362)	0.014	1.433 (0.919-2.234)	0.113	1.511 (1.035-2.206)	0.033	1.308 (0.839-2.040)	0.236
*IDH1* mutations	0.843 (0.466-1.527)	0.574			0.890 (0.492-1.611)	0.700		
*IDH2* mutations	1.113 (0.649-1.910)	0.697			0.987 (0.576-1.691)	0.963		
*TET2* mutations	0.953 (0.514-1.767)	0.879			0.945 (0.510-1.751)	0.857		
*RUNX1* mutations	1.853 (1.077-3.186)	0.026	2.169 (1.157-4.064)	0.016	1.644 (0.959-2.817)	0.071	1.742 (0.937-3.240)	0.079
*TP53* mutations	3.687 (2.144-6.339)	0.000	2.311 (1.187-4.497)	0.014	3.257 (1.912-5.549)	0.000	2.174 (1.128-4.189)	0.020

OS: overall survival; DFS: disease-free survival; HR: hazard ratio; CI: confidence interval; WBC: white blood cells. Variables in multivariate analysis including *TET3* expression (Low vs. High), age, WBC, karyotype (favorable vs. intermediate vs. poor), treatment regimen (with transplantation vs. without transplantation) and gene mutations (mutant vs. wild-type).

**Table 2:** Cox regression analyses of variables for OS and DFS in non-M3 AML patients.

**Variables**	**OS**				**DFS**			
	**Univariate analysis**		**Multivariate analysis**		**Univariate analysis**		**Multivariate analysis**	
	**HR (95% CI)**	***P***	**HR (95% CI)**	***P***	**HR (95% CI)**	***P***	**HR (95% CI)**	***P***
*TET3* expression	0.589 (0.403-0.862)	0.006	0.644 (0.425-0.975)	0.038	0.597 (0.408-0.873)	0.008	0.632 (0.422-0.945)	0.026
Age	1.033 (1.019-1.047)	0.000	1.011 (0.994-1.027)	0.203	1.027 (1.014-1.041)	0.000	1.012 (0.996-1.028)	0.136
WBC	1.001 (0.997-1.005)	0.609			1.001 (0.998-1.005)	0.450		
Karyotype risk	1.698 (1.308-2.205)	0.000	2.188 (1.592-3.008)	0.000	1.674 (1.292-2.169)	0.000	1.822 (1.356-2.448)	0.000
Treatment regimen	0.445 (0.311-0.636)	0.000	0.297 (0.195-0.453)	0.000	0.518 (0.363-0.740)	0.000	0.371 (0.246-0.559)	0.000
*FLT3* mutations	1.334 (0.903-1.969)	0.148	1.534 (0.953-2.469)	0.078	1.330 (0.902-1.963)	0.150	1.625 (1.032-2.558)	0.036
*NPM1* mutations	1.049 (0.717-1.535)	0.804			1.099 (0.751-1.608)	0.628		
*CEBPA* mutations	0.802 (0.407-1.581)	0.523			0.940 (0.477-1.852)	0.857		
*DNMT3A* mutations	1.414 (0.964-2.074)	0.077	1.520 (0.970-2.382)	0.068	1.329 (0.907-1.947)	0.144	1.362 (0.868-2.138)	0.179
*IDH1* mutations	0.735 (0.405-1.333)	0.311			0.778 (0.429-1.410)	0.408		
*IDH2* mutations	0.972 (0.566-1.671)	0.918			0.857 (0.499-1.471)	0.575		
*TET2* mutations	0.837 (0.451-1.554)	0.573			0.830 (0.447-1.542)	0.556		
*RUNX1* mutations	1.661 (0.965-2.860)	0.067	2.955 (1.580-5.678)	0.001	1.466 (0.854-2.515)	0.165	2.101 (1.139-3.874)	0.017
*TP53* mutations	3.214 (1.840-5.614)	0.000	2.578 (1.317-5.045)	0.006	2.818 (1.629-4.875)	0.000	2.239 (1.164-4.308)	0.016

OS: overall survival; DFS: disease-free survival; HR: hazard ratio; CI: confidence interval; WBC: white blood cells. Variables in multivariate analysis including *TET3* expression (Low vs. High), age, WBC, karyotype (favorable vs. intermediate vs. poor), treatment regimen (with transplantation vs. without transplantation) and gene mutations (mutant vs. wild-type).

**Figure 4:**
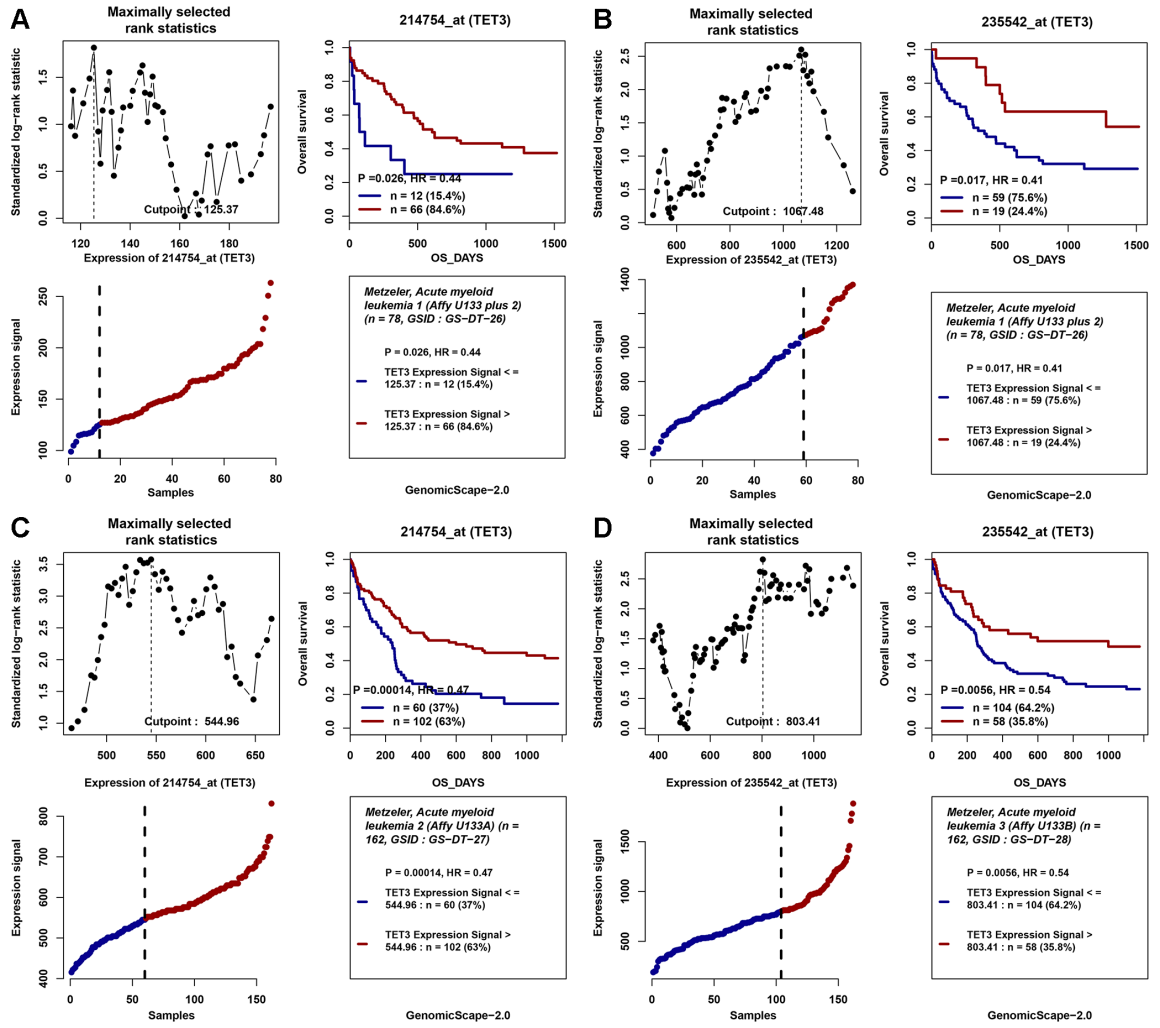
**The impact of *TET3* expression on overall survival of AML patients.** (**A**–**D**) Two independent cohorts of 162 and 78 cytogenetically normal AML (CN-AML) patients were obtained from Gene Expression Omnibus (GEO) data (http://www.ncbi.nlm.nih.gov/geo/; accession number GSE12417). Survival analysis was performed through the online web tool Genomicscape (http://genomicscape.com/microarray/survival.php). (**A**) probe 214754_at (TET3) in 78 CN-AML patients; (**B**) probe 235542_at (TET3) in 78 CN-AML patients; (**C**) probe 214754_at (TET3) in 162 CN-AML patients; (**D**) probe 235542_at (TET3) in 162 CN-AML patients.

In addition, the positive impact of high *TET3* expression on OS in cytogenetically normal AML (CN-AML) patients was also validated by Gene Expression Omnibus (GEO) data (GSE12417) via online web tool Genomicscape (Figure 4A–4D).

### Association between TET3 expression and clinical/molecular characteristics

Due to the significant association of *TET3* expression with AML prognosis, we next analyzed the clinical relevance of *TET3* expression with clinical/molecular characteristics in AML. As presented in Table 3. There were no significant differences between *TET3*^high^ and *TET3*^low^ groups in sex, age, white blood cells (WBC), bone marrow (BM)/peripheral blood (PB) blasts, and the distributions of cytogenetics (*P*>0.05). Significant difference was observed between two groups in the distribution of FAB subtypes (*P*=0.009). *TET3*^high^ patients was frequently occurred in FAB-M1/M4 (*P*=0.083 and 0.022, respectively), and less frequently occurred in FAB-M0 (*P*=0.016). Among common gene mutations, high expression of *TET3* was associated with *FLT3* wild-type and *NRAS* mutation (*P*=0.018 and 0.018, respectively). No significant differences were found between *TET3* expression with other gene mutations (*P*>0.05). Since *TET2* mutation is frequent molecular event in AML, we further analyzed the relationship between *TET2* mutation and *TET1/2/3* expression in AML patients. As presented in Supplementary Figure 1, no significant differences were found between *TET2* mutation (*TET2*^mu^) and *TET2* wild-type (*TET2*^WT^) regarding *TET1/2/3* expression (*P*>0.05).

**Table 3:** Correlation of TET3 expression with clinic-pathologic characteristics in AML.

**Patient's parameters**	***TET3* expression**		
	**Low (n=87)**	**High (n=86)**	***P***
Sex, male/female	44/43	48/38	0.543
Median age, years (range)	60 (21-88)	57 (18-82)	0.113
Median WBC, ×10^9^/L (range)	15.1 (0.5-297.4)	17 (0.4-223.8)	0.678
Median PB blasts, % (range)	45 (0-98)	29 (0-97)	0.370
Median BM blasts, % (range)	75 (32-100)	72 (30-100)	0.294
FAB classifications			**0.009**
M0	13	3	
M1	17	27	
M2	21	17	
M3	11	5	
M4	11	23	
M5	9	9	
M6	1	1	
M7	3	0	
No data	1	1	
Cytogenetics			0.637
normal	39	41	
t(15;17)	10	5	
t(8;21)	3	4	
inv(16)	3	7	
+8	5	3	
del(5)	1	0	
-7/del(7)	3	4	
11q23	1	2	
others	7	7	
complex	12	13	
No data	3	0	
Gene mutation			
FLT3 (+/-)	32/55	17/69	**0.018**
NPM1 (+/-)	22/65	25/61	0.611
DNMT3A (+/-)	24/63	18/68	0.376
IDH2 (+/-)	6/81	11/75	0.212
IDH1 (+/-)	7/80	9/77	0.611
TET2 (+/-)	8/79	7/79	1.000
RUNX1 (+/-)	7/80	8/78	0.794
TP53 (+/-)	8/79	6/80	0.782
NRAS (+/-)	2/85	10/76	**0.018**
CEBPA (+/-)	5/82	8/78	0.404
WT1 (+/-)	4/83	6/80	0.535
PTPN11 (+/-)	2/85	6/80	0.168
KIT (+/-)	3/84	4/82	0.720
U2AF1 (+/-)	2/85	5/81	0.278
KRAS (+/-)	3/84	4/82	0.720
SMC1A (+/-)	4/83	3/83	1.000
SMC3 (+/-)	3/84	4/82	0.720
PHF6 (+/-)	2/85	3/83	0.682
STAG2 (+/-)	2/85	3/83	0.682
RAD21 (+/-)	2/85	2/84	1.000

AML, acute myeloid leukemia; WBC, white blood cells; PB, peripheral blood; BM, bone marrow; FAB, French-American-British classification.

### TET3 expression may guide treatment choice between chemotherapy and HSCT

**Figure 5:**
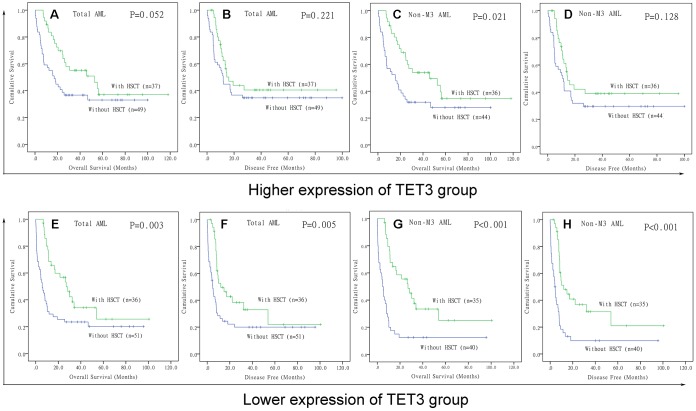
**The effect of hematopoietic stem cell transplantation on survival of AML patients among different *TET3* expression groups.** (**A**–**D**) Kaplan–Meier survival curves of overall survival and disease free survival in low *TET3* expression group. (**E**–**H**) Kaplan–Meier survival curves of overall survival and disease free survival in high *TET3* expression group.

Because low expression of *TET3* predicted poor clinical outcome in AML, we intended to investigate whether patients with low expression of *TET3* could benefit from hematopoietic stem cell transplantation (HSCT). We compared OS and DFS between patients with and without HSCT among both *TET3*^high^ and *TET3*^low^ groups. In *TET3*^high^ groups, although patients who received HSCT presented longer OS and DFS compared with patients who did not receive HSCT among both total AML (Figure 5A and 5B, *P*=0.052 and 0.221, respectively) and non-M3-AML (Figure 5C and 5D, *P*=0.021 and 0.128, respectively), the *P* did not attach statistical significance especially for DFS. However, in *TET3*^low^ groups, patients who accepted HSCT showed significantly longer OS and DFS than patients who did not accept HSCT among both total AML (Figure 5E and 5F, *P*=0.003 and 0.005, respectively) and non-M3-AML (Figure 5G and 5H, *P*<0.001 and 0.001, respectively).

### Correlations between TET3 expression and molecular signature

**Figure 6:**
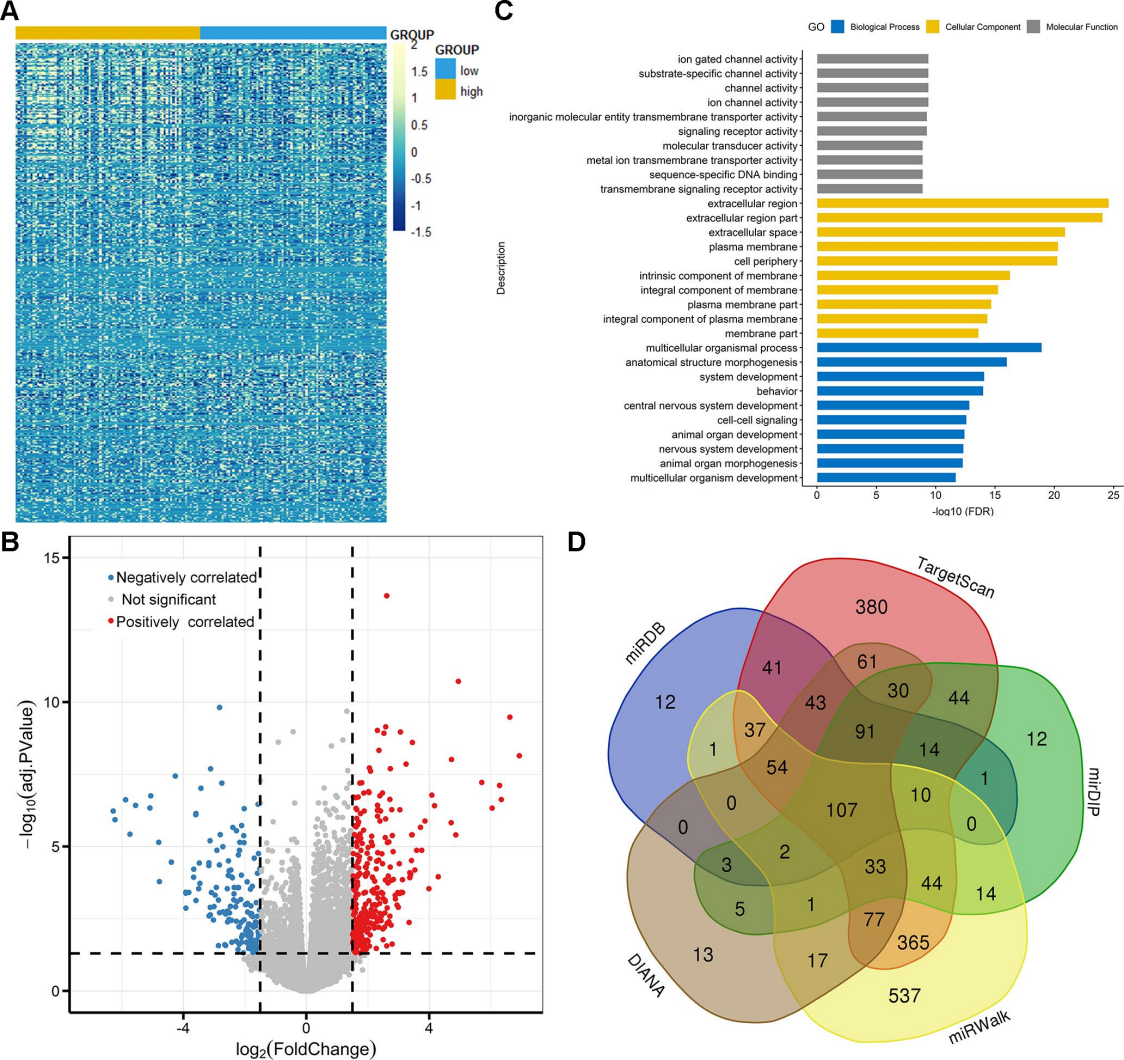
**Molecular signatures associated with *TET3* in AML.** (**A**) Expression heatmap of differentially expressed genes between *TET3*^low^ and *TET3*^high^ AML patients (FDR<0.05, *P*<0.05 and |log2 FC|>1.5). (**B**) Volcano plot of differentially expressed genes between *TET3*^low^ and *TET3*^high^ AML patients. (**C**) Gene Ontology analysis of DEGs conducted using online website of STRING (http://string-db.org). (**D**) Venn results of microRNAs which could target *TET3* predicted by DIANA (http://diana.imis.athena-innovation.gr/DianaTools/index.php? r=microT_CDS/index), miRDB (http://mirdb.org/miRDB/), mirDIP (http://ophid.utoronto.ca/mirDIP/), TargetScan (http://www.targetscan. org/vert_72/), and miRWalk (http://mirwalk.umm.uni-heidelberg.de/).

To gain insights into the biological function of *TET3* in AML, we first compared the transcriptomes of *TET3*^high^ and *TET3*^low^ groups. A total of 464 differentially expressed genes were identified (FDR<0.05, |log2 FC|>1.5; Figure 6A and 6B; Supplementary Table 2), in which 300 genes were positively correlated with *TET3* expression, and 164 were negatively correlated. Positively correlated genes such as *CDKN2B* and *ZIC2* were reported to have anti-leukemia effects [16, 17]. Among the negatively associated genes, several genes including *PAX2*, *IL2RA*, *SOX11*, and *PAK7* played as oncogenes in leukemia [18–21]. Furthermore, the Gene Ontology analysis was also showed in Figure 6C.

We next derived microRNA expression signatures associated with *TET3* expression, and only 5 microRNAs were significantly correlated (FDR<0.05, |log2 FC|>1.5; Supplementary Table 3). *MiR-196a-2* and *miR-1269* were positively correlated with *TET3* expression. Previous studies showed the anti-leukemia role of *miR-196a* as *ERG* regulators contributed to AML biology [22]. Negatively correlated microRNAs included *miR-1247*, *miR-205*, and *miR-935*. Interestingly, of these microRNAs, none of them were identified as predicted microRNAs that direct target *TET3* (Figure 6D, Supplementary Table 4).

## DISCUSSION

Aberrant promoter methylation, an important hallmark of cancer cells, is considered as a major mechanism underlying the activation/inactivation of tumor-related genes [1]. In addition to *DNMTs*, *TET* gene family encodes proteins that represent crucial factors in the active DNA demethylation pathway [3–5]. A loss-of-function mutation in the *TET2* gene is associated with leukemogenesis, drug response, and treatment outcome [11]. However, few studies investigated *TETs* expression and its clinical significance in AML [14, 15]. Herein, we systemically explored the *TETs* expression and its clinical significance in AML, and we hope that our findings could provide new insight into AML biology, improve treatment designs, and enhance the accuracy of prognosis for patients with AML. In this study, we showed that *TETs* expression showed differentially expressed in AML, which indicated different role of *TETs* during AML pathogenesis. In solid tumors, a number of studies showed the direct role of *TETs* in cancer biology. For example, two studies have showed that *TET1* was a tumor suppressor gene that inhibited colon cancer growth by derepressing inhibitors of the WNT pathway [23, 24]. Xu et al disclosed that tumor suppressive role of *TET2* promoted cancer immunity and immunotherapy efficacy [25]. Moreover, *TET2* controlled chemoresistant slow-cycling cancer cell survival and tumor recurrence [26]. Cui et al demonstrated that *TET3* as a potential tumor suppressor induced by the nuclear receptor *TLX* to regulate the growth and self-renewal in glioblastoma stem cells [27]. Moreover, several tumor suppressors, including *BTG2*, *TUSC1*, *BAK1*, *LATS2*, *FZD6* and *PPP2R1B*, were regarded as common targets of *TET3* [27]. Additionally, *TET3* expression was decreased in ovarian cancer tissues, acted as a suppressor of ovarian cancer by demethylating *miR-30d* precursor gene promoter to block TGF-β1-induced epithelial-mesenchymal transition [28]. In our study, we showed that *TET1* expression was significantly decreased in AML, whereas *TET2* and *TET3* expression was significantly increased in AML. Notably, we did not observe the direct association of *TET3* with these factors, and found that several tumor suppressor genes (*CDKN2B*, *ZIC2*, and *miR-196a*) and oncogenes (*PAX2*, *IL2RA*, *SOX11*, and *PAK7*) were associated with *TET3* in AML biology [16–22]. Moreover, these genes were important factors as cellular component or involving in many crucial biological processes contributing to cancer development. Lastly, *TET3* was differently expressed among the distributions of FAB subtypes in AML. These results suggested that the biological network of *TETs* in cancer was dependent on cancer type and stage specific.

Although previous studies showed the significant associations of *TET1* and *TET2* expression with AML prognosis [14, 15], herein, we only observed that *TET3* expression acted as an independent prognostic factor in AML, and could be overcame by HSCT. It was very interesting that *TET3* expression was increased in AML, and its high expression showed a positive effect in AML. Possible reason was that *TET3* expression may play a different role between cancer occurrence and development, and further functional studies are needed to explore the underlying mechanism in AML development. The expression pattern and clinical significance of *TET3* have been determined in several human cancers. Several studies revealed that high expression of *TET3* was revealed in renal cell carcinoma as well as endometrial cancers, and high mRNA levels of *TET3* were independent predictors of poor outcome in renal cell carcinoma patients [29, 30]; whereas, several other investigations reported that *TET3* was low-expressed in diverse human cancers. For instance, Bronowicka-Kłys et al showed that *TET3* transcript levels were lower in stage III samples of cervical cancer [31]. Moreover, *TET3* mRNA was decreased in chronic lymphocytic leukemia cells compared with healthy B cells [32]. In colorectal cancer, reduced transcript level of *TET3* was observed in cancerous tissue compared with their histopathologically unchanged counterparts [33]. In addition, Misawa et al reported that *TET3* methylation was highly associated with poor survival in T1 and T2 tumor stages of oropharyngeal cancer and oral cancer patients [34]. All these results further indicated that the role of *TET3* in diverse human cancers was specific among different cancer types.

In summary, our study demonstrated that *TETs* showed significant expression differences in AML, and *TET3* expression acted as a potential prognostic biomarker in AML, which may guide treatment choice between chemotherapy and HSCT.

## MATERIALS AND METHODS

### CCLE, HPA, and EMBL-EBI dataset

Firstly, *TETs* expression in human cancer cell lines is assessed by the CCLE dataset (https://www. broadinstitute.org/ccle), which provides public access to genomic data, analysis, and visualization for about 1000 cell lines [35]. Secondly, we also used The HPA dataset (https://www.proteinatlas.org/) to verify *TETs* expression in human cancer cell lines [36]. Lastly, *TETs* expression in AML cell lines is verified by the EMBL-EBI dataset (https://www.ebi.ac.uk), which has provided free and open access to a range of bioinformatics applications for sequence analysis since 1998 [37].

### GEPIA dataset

*TETs* expression in AML patients and normal controls was analyzed by the GEPIA web (http://gepia.cancer-pku.cn/), whose data from TCGA and the GTEx projects [38].

### Patients from TCGA and GEO

A total of 173 AML patients with available *TETs* expression data from TCGA (https://cancergenome.nih.gov/ and http://www.cbioportal.org/) were identified and included in this study [39]. Clinical and molecular characteristics were obtained, including, age, sex, WBC counts, PB blasts, BM blasts, FAB subtypes, and the frequencies of genetic mutations as presented in Table 3. After induction chemotherapy, consolidation treatment included chemotherapy (100 patients received) and HSCT (73 patients accepted).

In addition, two cohorts of 162 and 78 CN-AML patients from GEO data (GSE12417) were also included. The online web tool Genomicscape (http://genomicscape.com/microarray/survival.php) was applied to validate the prognostic value of *TETs* expression among CN-AML patients.

### Bioinformatics analysis

The details for the identification of microRNAs targeting *TET3* were reported as our previous study [40].

### Statistical analysis

Statistical analysis and figures creation were performed on SPSS 22.0 software. Mann-Whitney’s U test was used for the comparison of continuous variables, whereas Pearson Chi-square analysis or Fisher exact test was applied for the comparison of categorical variables. The prognostic effect of *TETs* expression on DFS and OS was evaluated analyzed though Kaplan-Meier analysis and Cox regression analysis. The two-tailed *P* value < 0.05 in all statistical analysis was defined as statistically significant.

### Ethical approval

All procedures performed in studies involving human participants were approved by the Ethics Committee of the Affiliated People’s Hospital of Jiangsu University and the Washington University Human Studies Committee and with the 1964 Helsinki declaration and its later amendments or comparable ethical standards. Informed consent was obtained from all patients included in this study.

## Supplementary Material

Supplementary Figure 1

Supplementary Table 1

Supplementary Table 2

Supplementary Table 3

Supplementary Table 4
