# Supplementary material for: Expression and prognosis analysis of TET family in acute myeloid leukemia
Source: Aging (Albany NY). 2020 Mar 25;12(6):5031–47. doi: 10.18632/aging.102928 (PMC7138570; doi:10.18632/aging.102928)
Supplement: Supplementary Figure 1 [file aging-12-102928-s005..pdf]

SUPPLEMENTARY FIGURE

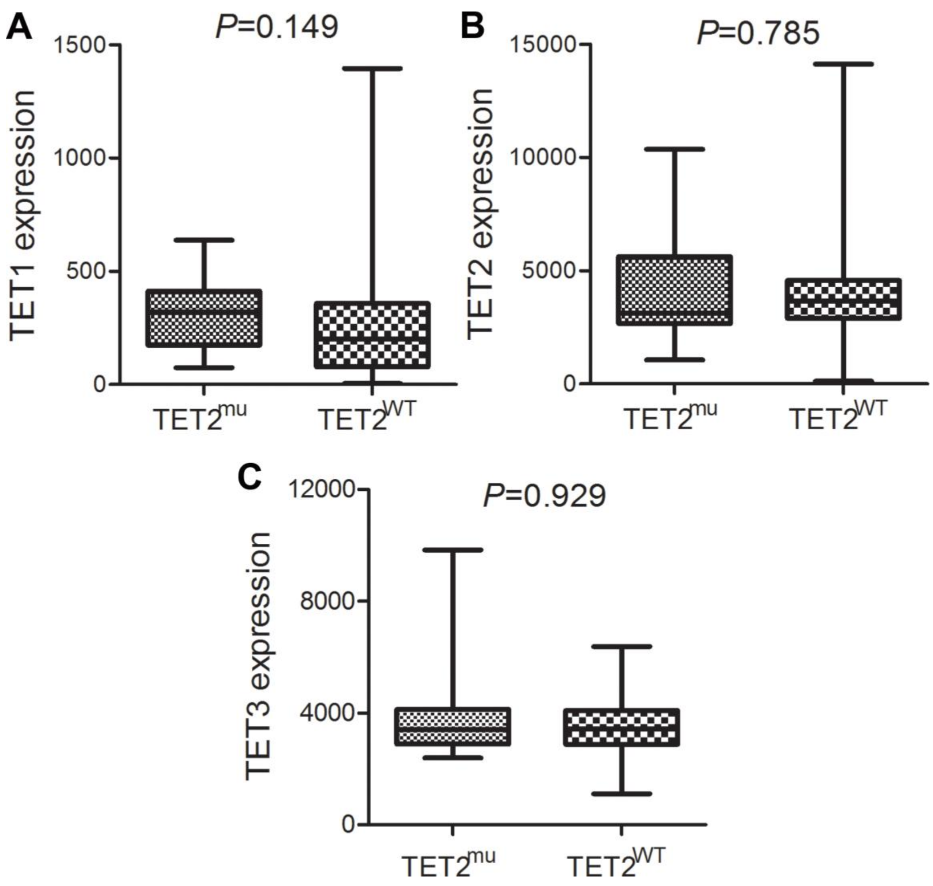

Supplementary Figure 1. The expression of *TETs* in AML patients with and without *TET2* mutation. (A): For *TET1* expression; (B): For *TET2* expression; (C): For *TET3* expression.
